# Supplementary material for: First Evidence Indicates the Physiology- and Axial-Myopia-Dependent Profiles of Steroid Hormones in Aqueous Humor
Source: Metabolites. 2022 Dec 5;12(12):1220. doi: 10.3390/metabo12121220 (PMC9787499; doi:10.3390/metabo12121220)
Supplement: Supplementary file 1 [file metabolites-12-01220-s001.zip › metabolites-2002783-supplementary.pdf]

Supplementary Materials

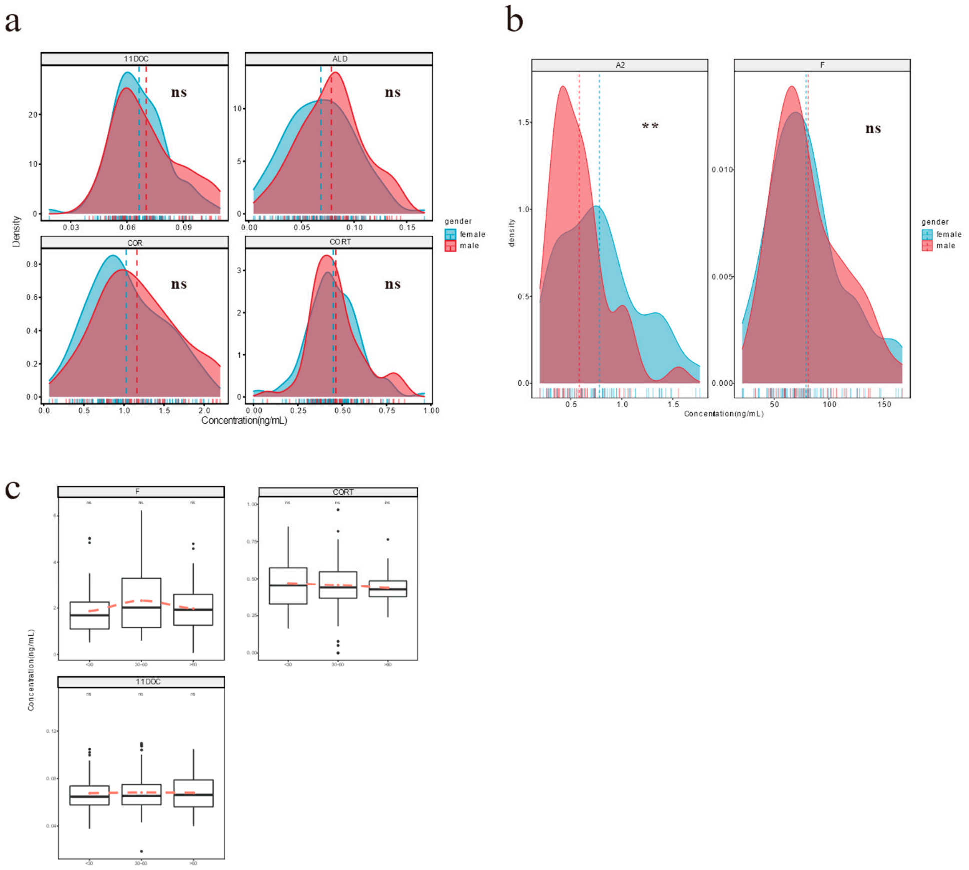

Figure S1. Steroid hormone distribution by age and sex. a) Steroid hormones distribution in males and females (ns represents the no significant difference). b) Steroid hormones (F and A2) distribution in males and females of plasma (** represents the p value <0.01, ns represents the no significant difference). .c) Steroid hormones among different age groups.

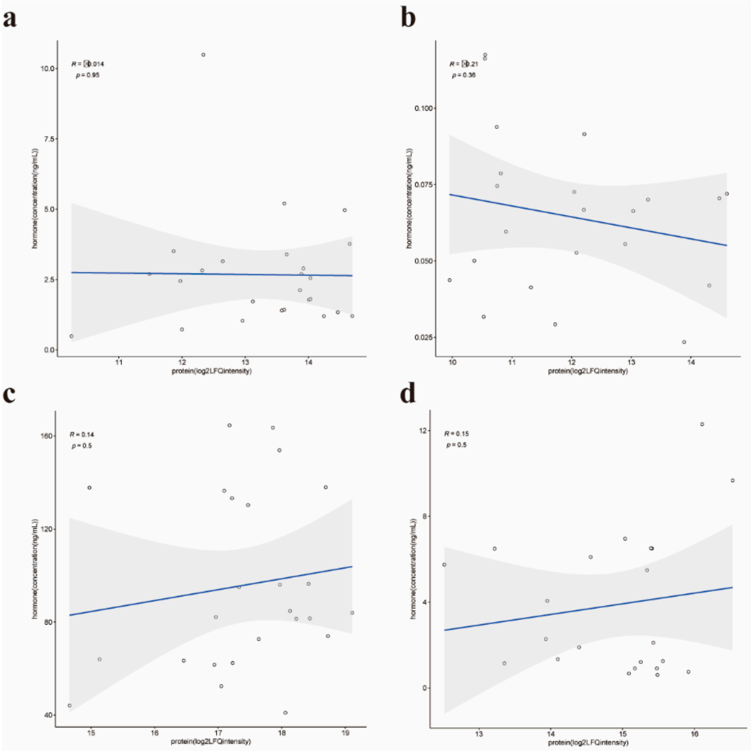

Figure S2 The abundance correlation of carrier proteins and steroid hormones. a) The abundance correlation plot for CBG and glucocorticoids in AHs. b) The abundance correlation plot for SHBG and sex hormones in AHs. c) The abundance correlation plot for CBG and glucocorticoids in plasmas. d) The abundance correlation plot for SHBG and sex hormones in plasmas.

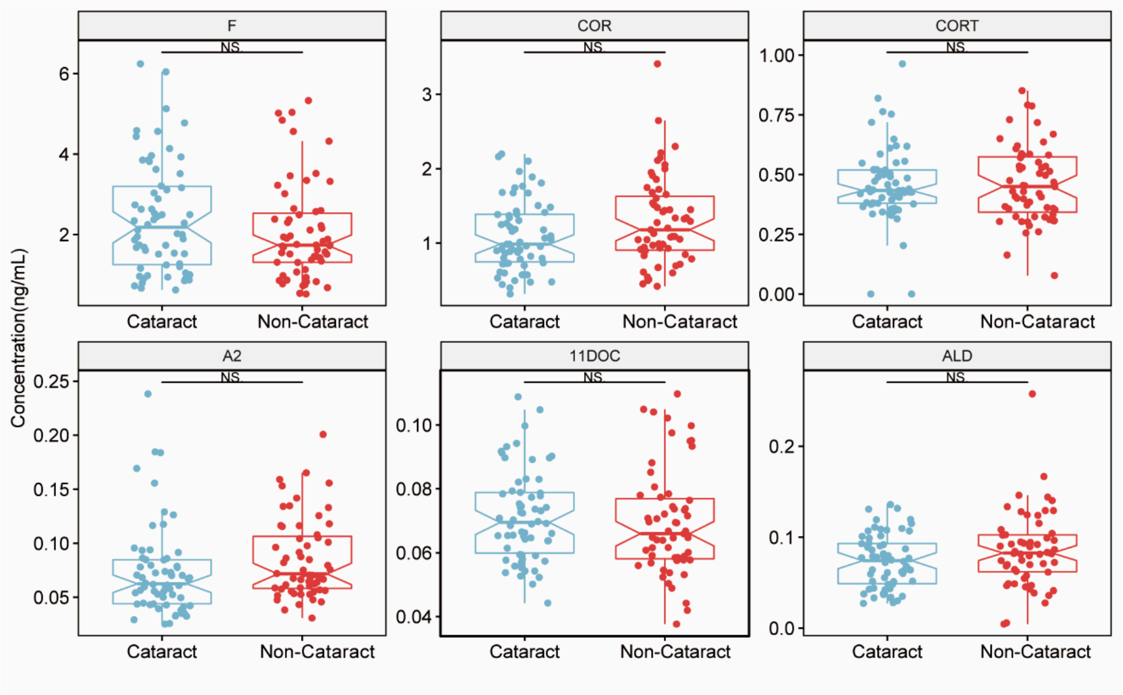

Figure S3 Box plot for steroid hormone concentrations in the cataract and normal groups (NS represents no significance difference)

Table S1. Clinical information of donors

Case Number	Case Group	Age	Gender	Eye	Diopter	Degree of Turbidity	AL (od mm)	AL (os mm)	Correspond Plasma
20201225P0037	ARC	73	female	od	-3.25DC*177	C4N4P3	22	21.8	yes
20201225P0002	ARC	85	female	od	-1.50DC*94	C3N3P2	23.27	23.24	yes
20201225P0005	ARC	53	female	od	-3.75DS-0.50DC*92	C2N3P4	22.24	22.11	yes
20201225P0067	ARC	79	male	od	-0.25DS-0.70DC*5	C2N3P2	23.74	23.82	yes
20201225P0149	ARC	68	female	os	-0.25DS-0.60DC*9	C2N3P2	23.07	23.05	yes
20201225P0041	ARC	69	female	od	-0.25DS-0.30DC*92	C3N2P2	22.34	22.52	yes
20201225P0012	ARC	84	male	od	-0.75DC*82	C3N4P3	23.58	23.56	yes
20201225P0034	ARC	77	male	od	-2.00DS-2.50DC*48	C2N3P2	24.2	23.84	yes
20201225P0003	ARC	82	male	od	-2.00DS-3.00DC*110	C2N3P2	24.11	24.29	yes
20201225P0152	ARC	73	male	os	+2.00DS-3.25DC*70	C4N3P3	24.16	23.95	yes
20201225P0137	ARC	91	male	os	-4.5DC*90	C4N4P4	22.54	22.56	yes
20201225P0121	ARC	65	female	os	+1.50DC*172	C3N2P2	23	23.12	yes
20200918P0020	ARC	67	female	od	-0.25DC*132	C2N2P2	24.62	24.28	yes
20200918P0013	ARC	71	female	od	+0.75DS+1.00DC*90	C2N3P2	23.4	23.66	yes
20200918P0030	ARC	76	male	od	-1.50DS-2.00DC*96	C3N3P3	23.12	23.41	yes
20200918P0019	ARC	73	male	od	+0.75DS+1.00DC*180	C3N3P3	24.29	24.17	yes
20200918P0014	ARC	77	female	os	-0.50DC*15	C2N3P2	23.39	23.55	yes
20200918P0032	ARC	74	male	od	-1.50DS	C3N3P3	23.39	23.33	yes
20200918P0044	ARC	78	male	od	-0.50DC*135	C4N4P2	23.56	23.7	
20200918P0046	ARC	50	male	os	-0.75DC*77	C3N3P3	23.33	23.58	
20201225P0015	ARC	61	female	od	-0.25DC*50	C2N3P2	22.01	22.1	
20201225P0147	ARC	80	female	os	-0.25DC*149	C3N3P2	24.6	24.72	
20201225P0072	ARC	69	female	os	+3.00DS-3.00DC*25	C4N4P3	24.54	24.8	
20201225P0140	ARC	62	female	od	+2.00DS	C2N2P2	22.56	22.61	
20201225P0160	ARC	61	male	os	-1.25DC*77	C2N3P2	24.11	23.92	
20201225P0033	ARC	83	male	os	-2.00DC*85	C4N3P3	23.3	23.27	
20201225P0043	ARC	64	female	os	-0.75DC*153	C2N3P2	23.66	23.63	
20201225P0095	ARC	66	male	od	+1.25DS+0.25DC*75	C3N3P2	23.99	23.88	
20201225P0009	ARC	63	female	od	+3.00DS	C2N3P2	23.74	23.32	
20201225P0071	ARC	80	male	od	-2.50DC*111	C2N3P2	22.22	22.8	yes
20201225P0019	DC	45	female	od	-0.50DC*160	C4N4P3	24.47	24.34	yes
20201225P0133	DC	79	female	od	-0.77DC*101	C2N3P2	22.43	22.33	yes
20201225P0065	DC	49	male	od	-1.75DS-0.75DC*85	C2N2P2	23.94	23.93	yes
20201225P0081	HM	27	male	od	-6.00DS-2.50DC*5		27.99	24.48	yes
20201225P0045	HM	30	female	od	-8.37DS-2.37DC*178		27.05	26.23	yes
20200918P0015	HM	30	female	od	-7.00DS-1.25DC*172		25.09	25.31	yes
20200918P0003	HM	27	male	od	-5.75DS		26.66	26.68	yes
20200918P0037	HM	27	female	od	-5.00DS-1.25DC*80		26.76	26.67	yes
20200918P0022	HM	36	female	od	-11.62DS-1.12DC*16		27.2	27.34	yes
20200918P0017	HM	26	female	od	-6.12DS-0.87DC*6		25.56	25.48	yes
20200918P0048	HM	21	female	od	-7.25DS-0.87DC*179		25.47	25.48	
20200918P0043	HM	20	male	od	-14.00DS-4.50DC*90		29.92	28.42	

20201225P0029	HM	36	female	od	-7.25DS		26.92	30.27	
20201225P0013	HM	30	female	od	-5.62DS-0.37DC*51		26.16	26.01	
20201225P0135	HM	25	female	od	-5.87DS-1.62DC*175		26.51	27.07	
20201225P0134	HM	23	female	od	-8.37DS-1.00DC*4		27.86	27.77	
20201225P0079	HM	45	female	od	-6.25DS-3.50DC*176		25.73	25.58	
20201225P0151	HM	36	male	od	-5.50DS		26.84	26.77	yes
20201225P0083	HM	19	male	od	-5.87DS-0.37DC*46		26	26	yes
20201225P0111	HM	28	female	od	-10.50DS		27.96	27.19	yes
20201225P0044	HM	31	female	od	-9.00DS-5.50DC*180		26.95	26.98	yes
20201225P0153	HM	32	female	od	-13.50DS		30.18	29.81	yes
20201225P0061	HM	24	female	od	-11.25DS-1.50DC*10		26	25.82	yes
20201225P0105	HM	25	female	od	-5.25DS		26.32	26.24	yes
20201225P0008	HM	26	female	od	-6.75DS-0.25DC*93		25.49	25.51	yes
20201225P0051	HM	39	female	od	-21.00DS-3.00DC*10		25.79	26.35	yes
20201225P0084	HM	33	male	od	-7.00DS		25.79	26.35	yes
20201225P0040	HM	18	male	od	-8.62DS-1.87DC*180		26.66	26.73	yes
20201225P0023	HM	35	female	od	-6		26	26	yes
20201225P0154	HM	28	male	od	-12.50DS-0.75DC*228		28.95	28.49	yes
20201225P0128	HM	23	female	od	-10.50DS		26	26	yes
20201225P0062	HM	23	male	od	-12.50DS-3.50DC*158		28.89	29.1	yes
20201225P0110	HM	23	female	od	-9.25DS-0.62DC*2		27	27.5	yes
20201225P0001	HM	28	female	od	-6.75DS		25.36	25.22	yes
20201225P0122	HM	28	male	od	-10.00DS-2.50DC*170		27.67	27.34	yes
20201225P0060	HM	19	male	od	-9.50DS-1.75DC*180		26	26	yes
20201225P0119	HM	23	female	od	-10.00DS-2.00DC*180		28.83	29.19	yes
20201225P0139	HM	26	male	od	-5.25DS-2.25DC*174		27.13	26.98	yes
20201225P0118	HM	42	female	od	-7.62DS-0.25DC*21		26.43	26.41	yes
20200918P0021	HM	24	female	od	-7.50DS-0.50DC*2		26	26	yes
20200918P0012	HM	27	female	od	-8.12DS-1.00DC*5		26.22	25.63	yes
20200918P0008	HM	24	female	od	-6.87DS-2.87DC*6		25.97	26.13	yes
20200918P0039	HM	27	female	od	-5.12DS-0.37DC*39		26.18	26.11	yes
20200918P0036	HMC	51	female	od	-8.00DS-0.75DC*110	C2N3P2	26.79	26.68	yes
20200918P0004	HMC	52	male	od	-16.00DS-0.75DC*4	C2N2P2	30.61	29.17	yes
20200918P0035	HMC	53	male	os	-8.50DS-1.50DC*95	C2N2P2	30.32	28.9	yes
20200918P0026	HMC	53	male	od	-11.00DS-2.00DC*70	C2N3P2	28.4	27.98	yes
20200918P0007	HMC	53	female	od	-7.50DS-1.00DC*90	C2N2P2	26.8	26.22	yes
20200918P0018	HMC	31	male	os	-9.25DS	C2N2P1	25.95	25.89	yes
20200918P0011	HMC	51	male	os	-10.00DS-0.50DC*25	C4N4P3	28.16	28.82	yes
20200918P0001	HMC	46	female	os	-9.00DS/-0.50DC*60	C2N2P2	26.63	25.77	yes
20200918P0005	HMC	50	female	od	-10.00DS	C2N2P2	28.21	28.67	yes
20200918P0028	HMC	42	male	os	-9.00DS-0.750DC*25	C3N2P3	25.36	27.7	yes
20200918P0034	HMC	47	male	os	-11.25DS-1.50DC*143	C2N2P2	28.9	28.15	yes
20200918P0038	HMC	44	female	od	-2.75DS	C2N2P2	25.89	25.7	yes
20200918P0002	HMC	54	male	os	-7.75DS-2.25DC*174	C2N2P2	27.22	27.6	yes

20200918P0016	LM	22	female	od	-5.87DS-0.50DC*2		25.89	25.83	yes
20200918P0047	LM	30	female	od	-5.50DS-0.25DC*2		24.89	24.88	
20201225P0066	LM	26	female	od	-4.50DS-1.00DC*10		24.78	25.02	
20201225P0014	LM	35	female	od	-4.75DS-1.12DC*150		24.6	24.16	
20201225P0097	LM	26	female	od	-7.00DS		24.45	24.46	
20201225P0059	LM	34	male	od	-4.50DS-0.37DC*136		25.32	25.37	
20201225P0021	LM	30	female	od	-5.00DS-1.50DC*60		24.96	24.4	
20201225P0098	LM	27	female	od	-4.12DS-0.87DC*13		24.91	24.38	
20201225P0126	LM	24	female	od	-5.50DS-1.00DC*175		26.08	25.83	
20201225P0068	LM	26	female	od	-4.50DS		25.14	25.09	
20201225P0017	LM	31	female	od	-5.25DS		25.31	25.34	
20201225P0082	LM	37	female	od	-3.75DS		23.99	23.93	
20201225P0016	LM	18	female	od	-5.50DS-1.50DC*2		25.72	26.12	
20201225P0091	LM	26	female	od	-3.50DS-1.00DC*171		24.08	24.65	yes
20201225P0093	LM	29	female	od	-5.50DS-0.62DC*87		25.27	25.77	yes
20201225P0094	LM	22	male	od	-7.75DS-1.25DC*8		24.99	24.61	yes
20200918P0027	LM	33	female	od	-5.00DS		23	23	yes
20200918P0031	ARC	68	male	od	-0.25DC*45	C2N3P3	23.78	23.33	yes
20200918P0040	ARC	60	male	os	-0.50DC*50	C2N2P2	24.63	24.78	yes
20201225P0030	ARC	66	male	od	+0.90DC*104	C3N3P2	24.03	23.6	yes
20201225P0052	ARC	73	female	od	-0.50DC*86	C3N4P2	22.67	22.86	yes
20201225P0116	ARC	83	male	os	-1.75DC*91	C2N3P3	23.58	23.58	yes
20201225P0108	ARC	84	female	od	-1.00DC*133	C3N4P2	22.62	22.52	yes
20201225P0085	ARC	73	female	od	-1.00DC*78	C2N3P2	22.6	22.69	yes
20201225P0109	ARC	89	male	od	-1.50DC*78	C3N3P3	24.35	24.04	yes
20201225P0039	ARC	53	female	os	-0.25DS-0.34DC*100	C1N3P3	23.9	23.66	yes
20201225P0022	ARC	75	female	os	+1.25DS+1.50DC*170	C2N3P2	23.26	23.29	yes
20201225P0101	ARC	65	male	od	-0.25DS-0.25DC*67	C2N4P2	23.46	23.2	yes
20200918P0051	ARC	68	female	od	-1.50DC*45	C4N4P4	23.71	23.49	
20201225P0115	ARC	91	female	os	-1.50DC*92	C4N4P4	23.44	22.95	
20201225P0114	ARC	82	female	od	-0.50DC*45	C4N3P3	23.4	23.38	
20201225P0027	HM	28	female	od	-6.37DS-0.87DC*2		26.64	26.56	
20201225P0092	HM	27	male	od	-11.00DS-3.00DC*5		29.61	28.36	
20201225P0130	HM	31	female	od	-8.37DS-1.25DC*2		28.64	27.67	
20201225P0078	HM	21	male	od	-14.50DS-0.25DC*7		29.32	28.78	
20201225P0018	HM	26	female	od	-10.00DS-0.25DC*25		28.38	28.17	
20201225P0035	HM	23	male	od	-8.00DS-3.00DC*175		29.12	28.76	yes
20200918P0029	HM	49	female	od	-10.25DS		26	26	yes
20200918P0041	HM	26	male	od	-7.50DS-1.75DC*175		27.31	27.43	yes
20201225P0049	HM	20	female	od	-7.25DS-1.25DC*38		27.92	25.17	yes
20201225P0020	HM	39	female	od	-4.75DS		26.71	26.74	yes
20201225P0090	HM	40	male	od	-4.75DS		26.49	26.67	yes
20201225P0142	HMC	58	male	od	-5.50DS-0.50DC*90	C2N2P2	25.64	25.67	yes
20201225P0129	HMC	54	male	od	-4.25DS-3.00DC*170	C2N3P3	26.53	26.15	yes

20200918P0045	HMC	52	male	os	-5.00DS	C1N1P4	26.74	26.51	
20200918P0050	HMC	72	female	od	-15.00DS-0.50DC*85	C3N4P2	29.72	26.94	
20201225P0155	HMC	57	male	os	-7.00DS-1.00DC*75	C2N2P2	27.14	26.92	
20201225P0103	HMC	37	male	od	-25.75DS	C2N2P3	33.8	25.74	
20201225P0031	HMC	59	female	os	-9.25DS-0.50DC*16	C3N2P2	28	29.17	
20201225P0024	HMC	55	female	od	-9.00DS-0.25DC*25	C3N4P2	28.05	25.34	
20201225P0074	HMC	52	male	od	-8.50DS -0.75DC 167	C2N2P2	27.75	24.6	
20201225P0010	HMC	41	male	od	-8.00DS-1.00DC*5	C2N2P2	26.05	26.12	
20201225P0146	HMC	58	female	os	-16.00DS-1.50DC*121	C2N4P4	27.17	30.06	
20201225P0080	HMC	62	male	os	-7.50DS-1.00DC*75	C2N2P2	25.79	26.32	
20201225P0026	HMC	68	female	os	-7.00DS-1.50DC*175	C3N2P2	27.06	25.73	
20201225P0050	HMC	74	male	os	-20.00DS-1.50DC*155	C2N2P2	31.31	31.31	
20201225P0028	HMC	48	male	os	-14.25DS-2.25DC*80	C2N3P2	27.42	27.48	
20201225P0025	HMC	76	female	os	-7.50DS-0.75DC*80	C2N2P2	27.6	28.15	
20201225P0100	HMC	54	female	os	-23.00DS	C2N2P2	32.49	31.5	yes
20201225P0124	HMC	75	male	os	-12.00DS-2.50DC*65	C3N3P2	28.98	28.99	yes
20201225P0104	LM	31	female	od	-2.75DS		24.57	24.47	yes
20201225P0148	LM	27	female	od	-5.00DS		25.5	25.37	yes
20201225P0141	LM	28	female	od	-5.25DS-2.00DC*5		25.07	25.96	yes
20201225P0058	LM	24	male	od	-3.25DS		25.36	25.39	yes
20201225P0159	LM	36	female	od	-5.00DS		25.61	25.47	yes
20201225P0088	LM	30	female	od	-4.75DS		24.81	24.85	yes
20201225P0145	LM	23	female	os	-3.75DS		25.86	25.86	yes
20201225P0158	LM	24	female	od	-5.25DS		24.41	24.51	yes
20201225P0138	LM	24	female	od	-5.00DS		23	23	yes
20200918P0025	LM	31	female	od	-5.25DS-1.00DC*12		24.68	24.12	yes
20200918P0006	HM	20	male	od	-10.87DS-4.37DC*1		30.9	27.88	yes
20200918P0024	HMC	37	female	od	-23.00DS	C1N1P3	32.04	30.84	yes
20200918P0023	HMC	41	female	od	-25.00DS-1.50DC*162	C3N4P2	33.64	32.41	yes
20200918P0033	HMC	60	male	od	-21.50DS	C3N3P2	28.68	29.31	
20200918P0042	HMC	49	female	od	-24.00DS-2.25DC*135	C2N2P2	32.6	31.87	
20201225P0069	HMC	51	male	od	-29.00DS	C2N2P2	31.48	30.97	
20201225P0156	HMC	53	female	os	-20.00DS-1.00DC*70	C2N2P2	31.49	31.17	
20201225P0064	HMC	46	male	od	-14.00DS	C2N2P2	28.8	28.56	
20201225P0113	HMC	58	female	od	-6.00DS	C3N2P3	26.34	25.74	
20201225P0125	HMC	69	male	os	-8.5DS-1.50DC*85	C2N2P2	31.42	30.18	
20201225P0099	HMC	54	male	od	-15.50DS-1.50DC*20	C2N3P2	25.93	25.96	
20201225P0004	HMC	52	female	os	-15.00DS-0.50DC*7	C2N2P2	29.07	30.06	
20201225P0144	HMC	50	female	os	-3.00DS	C2N3P2	26.83	26.73	
20201225P0057	HMC	55	male	od	-11.00DS	C2N2P2	31.18	32.42	
20201225P0165	HMC	63	male	os	-22.50DS	C5N4P2	28.94	28.85	

Table S2. Multiple reaction monitoring transitions of steroid hormones

Steroid Hormone	IS	Mode	Quantitative	Parent transition (m/z)	Product transition (m/z)	Declustering Potential (Volt)	Collision Energy (Volt)
17OHPr	no	neg	yes	331.2	287.1	-55	-28
17OHPr	no	neg		331.2	313.3	-55	-28
17OHPr	yes	neg		335.2	316.3	-60	-28
DHEAS	no	neg	yes	367.3	79.9	-90	-42
DHEAS	yes	neg		373.2	80.1	-120	-135
E1	no	neg	yes	269.1	145	-150	-48
E1	no	neg		269.1	143	-150	-38
E1	yes	neg		273.1	147	-90	-53
E2	no	neg		271.1	183.1	-170	-54
E2	no	neg	yes	271.1	145	-170	-51
E2	yes	neg		274.1	185	-170	-52
E3	no	neg		287.3	143.3	-130	-65
E3	no	neg	yes	287.3	171.3	-130	-47
E3	yes	neg		290.1	174	-170	-47
11DOC	no	pos	yes	347.11	109	115	40
11DOC	no	pos		347.11	97	115	38
11DOC	yes	pos		352.2	100.1	130	31
17OHP	no	pos	yes	331	97.1	120	27
17OHP	no	pos		331	109.1	130	46
17OHP	yes	pos		334.2	100	100	30
21DOC	no	pos	yes	347.3	311.1	80	18
21DOC	no	pos		347.3	269.4	80	25
21DOC	yes	pos		355.2	180	60	28
A2	no	pos	yes	287.112	97.1	100	28
A2	no	pos		287.112	109	100	36
A2	yes	pos		290.2	100.1	71	35
ALD	no	pos	yes	361.1	343	110	25
ALD	no	pos		361.1	315.2	110	28
ALD	yes	pos		368.2	350.2	76	25
COR	no	pos	yes	361.3	163.3	120	31
COR	no	pos		361.3	121	120	39
COR	yes	pos		368.2	169.1	76	35
CORT	no	pos		347.2	121	80	32
CORT	no	pos	yes	347.2	329.1	80	20
CORT	yes	pos		355.3	337.2	90	22
DHEA	no	pos	yes	271.183	213.1	100	24
DHEA	no	pos		271.183	197.3	100	27
DHEA	yes	pos		277.2	219.3	75	24
DHT	no	pos	yes	291.2	255.2	90	22
DHT	no	pos		291.2	273.1	90	22
DHT	yes	pos		294.3	258.2	80	22

DOC	no	pos	yes	331.2	109	80	31
DOC	no	pos		331.2	96.8	100	26
DOC	yes	pos		339.3	100	100	29
F	no	pos		363.13	121.1	120	41
F	no	pos	yes	363.13	327.3	120	25
F	yes	pos		367.2	121.1	66	35
P	no	pos	yes	315.1	97.1	120	25
P	no	pos		315.1	109.1	120	30
P	yes	pos		318.2	100.1	95	50
Pr	no	pos	yes	299.2	281.3	100	22
Pr	no	pos		299.2	159.2	100	30
Pr	yes	pos		321.3	285.2	40	16
T	no	pos	yes	289.135	97	100	30
T	no	pos		289.135	109	100	34
T	yes	pos		292.2	100	90	30

Table S3 The linear and limit of quantification in LC MS/MS for steroid hormones

Steroid hormone	Actual Concentration(ng/mL)	Mean(ng/mL)	SD	CV (%)	SNR	R2
F	0.06	0.061	0.0049	8%	22	0.991
COR	0.1	0.095	0.0096	10%	147	0.992
11DOC	0.006	0.008	0.0015	21%	13	0.992
17OHP	0.02	0.011	0.0013	12%	18	0.994
ALD	0.005	0.006	0.0002	3%	17	0.991
A2	0.003	0.002	0.0001	3%	4	0.991
DHEA	0.03	0.032	0.0030	10%	71	0.990
T	0.01	0.010	0.0009	9%	41	0.997
17OHPr	0.32	0.301	0.0284	9%	23	0.996
21DOC	0.05	0.052	0.0033	6%	6	0.990
DHEAS	1	0.902	0.0456	5%	9	0.991
E3	0.02	0.019	0.0013	7%	4	0.997
Pr	1.25	1.164	0.0305	3%	12	0.992
P	0.01	0.010	0.0005	5%	27	0.996
DOC	0.005	0.005	0.0002	3%	14	0.996
E2	0.02	0.021	0.0030	14%	7	0.995
E1	0.5	0.574	0.0414	7%	77	0.990
CORT	0.03	0.031	0.0015	5%	18	0.995
DHT	0.08	0.071	0.0024	3%	27	0.991

Table S4 The reproducibility in intra- or inter-assays

Compound	Level	Expected Concentration (ng/ml)	within -run				between- run			
			mean(ng/ml)	SD	CV(%)	accuracy	mean(ng/ml)	SD	CV(%)	accuracy
11DOC	low	0.04	0.04	0.0022	6%	-1%	0.04	0.0021	5%	0%
11DOC	high	5.00	4.99	0.3368	7%	0%	4.93	0.2880	6%	-1%
17OHP	low	0.10	0.10	0.0053	5%	-1%	0.10	0.0051	5%	2%
17OHP	high	12.50	12.42	0.0994	1%	-1%	12.49	0.2126	2%	0%
17OHPr	low	0.31	0.34	0.0057	2%	9%	0.34	0.0194	6%	8%
17OHPr	high	40.00	40.24	3.2788	8%	1%	39.69	2.8400	7%	-1%
21DOC	low	0.20	0.19	0.0072	4%	-3%	0.19	0.0141	7%	-1%
21DOC	high	25.00	24.82	1.3845	6%	-1%	24.72	1.0668	4%	-1%
A2	low	0.04	0.04	0.0035	9%	1%	0.04	0.0034	9%	1%
A2	high	5.00	4.98	0.1715	3%	0%	4.97	0.1404	3%	-1%
ALD	low	0.02	0.02	0.0013	7%	-4%	0.02	0.0015	8%	-1%
ALD	high	2.50	2.57	0.2237	9%	3%	2.39	0.2645	11%	-4%
COR	low	0.39	0.40	0.0386	10%	1%	0.39	0.0269	7%	1%
COR	high	50.00	51.54	3.9006	8%	3%	50.34	3.1974	6%	1%
CORT	low	0.12	0.12	0.0077	7%	-1%	0.11	0.0087	8%	-5%
CORT	high	15.00	16.09	0.9567	6%	7%	15.65	0.8692	6%	4%
DHEA	low	0.12	0.13	0.0032	3%	10%	0.13	0.0038	3%	10%
DHEA	high	15.00	14.59	1.2189	8%	-3%	15.19	1.1425	8%	1%
DHEAS	low	31.25	32.58	2.3529	7%	4%	30.73	2.6831	9%	-2%
DHEAS	high	4000.00	4069.69	319.3828	8%	2%	4157.46	278.1861	7%	4%
DHT	low	0.08	0.07	0.0046	6%	-4%	0.08	0.0052	7%	-2%
DHT	high	10.00	9.67	0.9225	10%	-3%	10.23	1.0807	11%	2%
DOC	low	0.02	0.02	0.0017	9%	0%	0.02	0.0015	8%	-3%
DOC	high	2.50	2.49	0.0743	3%	0%	2.51	0.0698	3%	0%
E1	low	0.50	0.55	0.0441	8%	11%	0.57	0.0406	7%	14%
E1	high	4.00	3.67	0.2191	6%	-8%	3.69	0.1564	4%	-8%
E2	low	0.08	0.08	0.0097	13%	-1%	0.08	0.0099	12%	5%
E2	high	10.00	10.20	0.1842	2%	2%	10.15	0.2564	3%	1%
E3	low	0.04	0.04	0.0047	11%	9%	0.08	0.0109	14%	4%
E3	high	5.00	4.83	0.4843	10%	-3%	5.04	0.5181	10%	1%
F	low	0.98	1.03	0.0415	4%	6%	1.04	0.0341	3%	7%
F	high	125.00	127.59	2.2245	2%	2%	127.90	2.8778	2%	2%
P	low	0.08	0.08	0.0026	3%	-3%	0.08	0.0063	8%	0%
P	high	10.00	9.97	0.4228	4%	0%	9.92	0.3287	3%	-1%
Pr	low	1.25	1.23	0.0796	6%	-2%	1.24	0.0778	6%	-1%
Pr	high	40.00	40.73	2.4441	6%	2%	41.88	8.3702	20%	5%
T	low	0.08	0.07	0.0024	3%	-5%	0.08	0.0045	6%	-3%
T	high	10.00	9.99	0.2458	2%	0%	10.05	0.1957	2%	1%

Table S5 The average concentration of steroid hormones in AHs and plasma

Compound	plasma (n=107)		AH (n=107)	
	Mean (ng/mL)	SD	Mean (ng/mL)	SD
DOC	0.036	0.029	/	/
E2	0.063	0.071	/	/
E1	0.166	0.020	/	/
DHT	0.248	0.267	/	/
11DOC	0.326	0.318	0.068	0.016
21DOC	0.57	0.42		
ALD	0.642	0.194	0.073	0.032
A2	0.704	0.360	0.073	0.039
17OHP	1.037	0.932	/	/
P	1.670	3.901	/	/
T	1.827	2.242	/	/
17OHPr	2.125	2.497	/	/
Pr	2.703	1.401	/	/
CORT	2.831	2.197	0.455	0.147
DHEA	3.234	2.049	/	/
COR	17.200	5.258	1.082	0.481
F	79.859	33.245	2.102	1.225
DHEAS	357.471	218.308	/	/
